# Nutritional Status in Chinese Patients with Obesity Following Sleeve Gastrectomy/Roux-en-Y Gastric Bypass: A Retrospective Multicenter Cohort Study

**DOI:** 10.3390/nu14091932

**Published:** 2022-05-05

**Authors:** Chunlan Zhang, Xi Chen, Shiping Liu, Wei Liu, Dalong Zhu, Xiaoying Li, Shen Qu, Zhiming Zhu, Jingjing Zhang, Zhiguang Zhou

**Affiliations:** 1 National Clinical Research Center for Metabolic Diseases, Metabolic Syndrome Research Center, Key Laboratory of Diabetes Immunology, Ministry of Education, Department of Metabolism and Endocrinology, The Second Xiangya Hospital of Central South University, Changsha 410011, China; 198201033@csu.edu.cn (C.Z.); chenxi0206@csu.edu.cn (X.C.); shipingliu119@csu.edu.cn (S.L.); 2 Department of Metabolic Surgery, Department of Biliopancreatic Surgery, The Second Xiangya Hospital, Central South University, Changsha 410011, China; liuweixy@csu.edu.cn; 3 Department of Endocrinology, Drum Tower Hospital Affiliated to Nanjing University Medical School, Nanjing 210000, China; zhudalong@nju.edu.cn; 4 Department of Endocrinology and Metabolism, Zhongshan Hospital, Fudan University, Shanghai 200000, China; li.xiaoying@zs-hospital.sh.cn; 5 Department of Endocrinology and Metabolism, School of Medicine, Shanghai Tenth People’s Hospital, Tongji University, Shanghai 200000, China; qushencn@hotmail.com; 6 Chongqing Hypertension Institute, Department of Hypertension and Endocrinology, Daping Hospital, The Third Military Medical University, Chongqing 400000, China; zhuzm@tmmu.edu.cn

**Keywords:** metabolic surgery, obesity, nutrition, anemia, bone mineral density

## Abstract

Metabolic surgery (MS) is one of the most effective therapies for treating obesity. Due to the lack of multicenter cohort research on nutritional evaluations after surgery in Chinese patients, we explored the changes in nutritional status following MS in Chinese patients. This was a retrospective study of patients (*n* = 903) who underwent sleeve gastrectomy (SG) (*n* = 640) or Roux-en-Y gastric bypass (RYGB) (*n* = 263) for obesity at five different hospitals in China between 17 February 2011, and 20 December 2019. Major nutrients were evaluated at baseline and 1, 3, 6, and 12 months postoperatively. Hb levels decreased, and anemia prevalence increased at 12 months after MS in the premenopausal female group. Moreover, patients with preoperative anemia had an increased risk of postoperative anemia. The ferritin levels (*p* < 0.001) decreased and iron deficiency increased (*p* < 0.001) at 12 months after MS among premenopausal females. No significant changes in folate deficiency and vitamin B12 deficiency were found throughout the study. The bone mineral density (BMD) of the femoral neck, lumbar spine, and total hip significantly decreased from baseline to 12 months after MS; however, no new patients developed osteopenia or osteoporosis after MS. Based on 12 months of follow-up, premenopausal females presented a high incidence of anemia after MS. Although we found no differences in osteopenia and osteoporosis prevalence after MS, the BMD did decrease significantly, which suggests that nutrient supplements and long-term follow-up are especially necessary postoperation.

## 1. Introduction

Obesity is highly associated with type 2 diabetes (T2D), cardiovascular disease, and even some cancers and has become a major public health issue in China [1,2]. Based on Chinese criteria for obesity, 51% of Chinese adults were either overweight or obese according to the current study [3]. Metabolic surgery (MS) is the most efficient treatment for severe obesity and related diseases and is associated with the maintenance of long-term positive effects on weight and metabolic control [4,5,6]. The beneficial results of MS come with a cost; one such is postoperative nutritional complications. After years of practice and long-term follow-up, multiple nutritional deficiencies, such as anemia, bone demineralization, hypoproteinemia, and vitamin and trace element deficiencies, were reported in patients in Western countries following MS [7,8,9,10,11]. Patients showed a higher rate of anemia has been reported following gastric bypass in Western countries, which can be commonly caused by iron, vitamin B12, and folate deficiency, all of which may be affected by surgery [12,13,14]. In addition, nutrient deficiencies were severer in obese patients than normal-weight individuals prior to MS, and this is definitely further aggravated by MS [15,16,17,18]. A study highlighted that for any given BMI, Asians have a greater prevalence of diabetes than Europeans [19]. Thus, the body mass index (BMI) thresholds for MS are decreased by 2.5 kg/m^2^ for Asian patients compared with patients from Western countries [20].

At present, a few MS studies with a small number of patients have included outcomes related to micronutrient deficiencies in China [21,22]. These results impel us to conduct further studies on the adverse effects of MS on the nutritional status of obese subjects in China. Our study is the first multicenter clinical study and has the largest population focused on the impact of nutritional deficiencies in Chinese subjects after MS.

## 2. Materials and Methods

### 2.1. Participants

**Figure 1:**
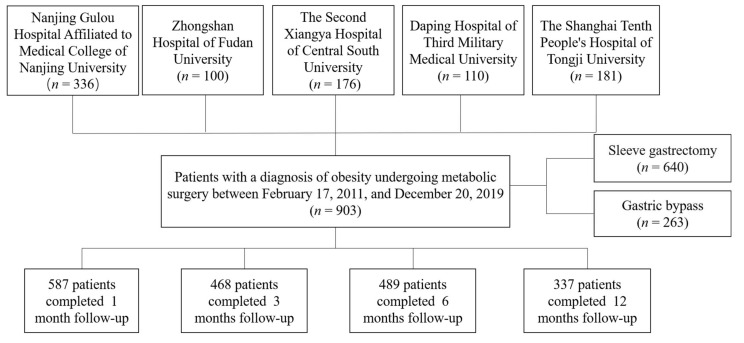
Flow chart of the study.

Nine hundred and three obese patients were recruited from five different hospitals in China (Figure 1). All subjects accepting SG or RYGB met the criteria for MS [20]. T2D and NGT (normal glucose tolerance) was diagnosed according to the World Health Organization criteria [23]. After surgery, patients were advised to take supplemental multivitamins, including iron 45–60 mg (daily), vitamin B12 350–500 mg (daily), folate 400–1000 mg (daily), and vitamin D3 3000 IU (daily), calcium 1200–1500 mg (daily) and protein 60–120 g (daily) [24]. Moreover, patients were asked to complete the follow-up evaluation at 1, 3, 6, and 12 months for the first postsurgical year.

### 2.2. Study Design and Laboratory Analysis

Anthropometric data were recorded by the study physicians, before and 1 month, 3 months, 6 months, and 12 months after the surgery. Venous blood samples were tested for blood sugar, blood fat, C peptide (CP), complete blood count, albumin, vitamin B12, vitamin D, ionized calcium and folate, ferritin, and iron. Dual-energy X-ray absorptiometry (DEXA) was used to measure BMD. The percentage of excess BMI loss was defined as (100 × [baseline BMI − follow-up BMI]/[baseline BMI − 25]).

### 2.3. Definitions of Anemia and Nutrition Deficiencies

Anemia was defined as a hemoglobin (Hb) level <130 g/L in men and <120 g/L in women, and anemia severity was classified as mild (Hb > 110 g/L), moderate (Hb between 80 and 110 g/L), and severe (Hb < 80 g/L) [25]. Vitamin B12 deficiency and folate deficiency were defined as serum levels <203 pg/mL and <4 ng/mL, respectively [26]. Iron deficiency was defined as a ferritin level <30 ng/mL [27]. Vitamin D deficiency was defined as a 25-OH vitamin D level of <20 ng/mL, and vitamin D insufficiency was defined as a 25-OH vitamin D level of 21–29 ng/mL [28]. The diagnosis of osteoporosis in postmenopausal women and older men is a T-score of   ≤−2.5 at the lumbar spine, femur neck, or total hip by BMD testing, and that of osteopenia was a T-score of <−1 and >−2.5 [29].

### 2.4. Statistical Analysis

The data are presented as the mean ± standard error (SD) for normally distributed parameters or as the median (25–75th percentile) for non-normally distributed parameters. Continuous variables with normal distribution were compared by the *t*-test for unpaired samples and paired *t*-test for paired samples. Unpaired samples with a non-normal distribution were compared by the nonparametric Mann–Whitney test and paired samples were compared by the Wilcoxon signed-rank test. To compare changes from the baseline to the each time points, we used repeated-measures analysis of variance (ANOVA) with Bonferroni post hoc testing. For binary variable samples, we used McNemar tests to analyze the differences among the paired time points. The chi-square method or Fisher’s exact test was used to compare the differences between categorical variables. A case-matched analysis (1:1) was performed to compare the effectiveness between SG and RYGB. The patients were matched by sex, age (±2 years), BMI (±1 kg/m^2^), and HbA1c (±0.5%). Statistical significance was defined as *p* < 0.05. For the statistical analyses, we used SPSS version 23.0 (IBM Corporation, Chicago, IL, USA) software. A two-tailed *p* < 0.05 was considered statistically significant.

## 3. Results

### 3.1. SG and RYGB Are Comparatively Effective with Respect to Weight Loss and Glycemic Control for Patients with Obesity

A total of 903 patients who accepted MS in the form of SG (*n* = 640) or RYGB (*n* = 263) were included in this study. The patients’ preoperative characteristics are shown in Table 1. Compared to patients who underwent RYGB, patients who underwent SG were younger, had a higher preoperative BMI, and had mild metabolic disorders. The alterations in the weight and metabolic parameters during the 1-year follow-up are shown in Supplementary Table S1. BMI, glucose metabolism, lipid profile, and blood pressure improved significantly after MS.

Patients who completed all the follow-up time points were included in further analyses. To exclude biases related to age, preoperative BMI, and blood glucose on weight loss and metabolic control, we further performed a case-matched study (1:1 proportion) based on sex, age (±2 years), BMI (±1 kg/m^2^), and HbA1c (hemoglobin A1c) (±0.5%). A total of 98 pairs of SG and 98 pairs of RYGB were matched and further analyzed. As shown in Table 2, patients in the RYGB group had an excessive LDL-C (low-density lipoprotein cholesterol) decrease (2.2 ± 0.5 vs. 2.7 ± 1.0, *p* = 0.008) compared to the SG group at the 1-year follow-up. Moreover, there was no significant difference regarding weight loss or glycemic and blood pressure control at the 1-year follow-up after surgery between SG and RYGB. These data indicated that RYGB exhibited a superior effect on lipid disorders compared to SG.

### 3.2. Anemia

**Figure 2:**
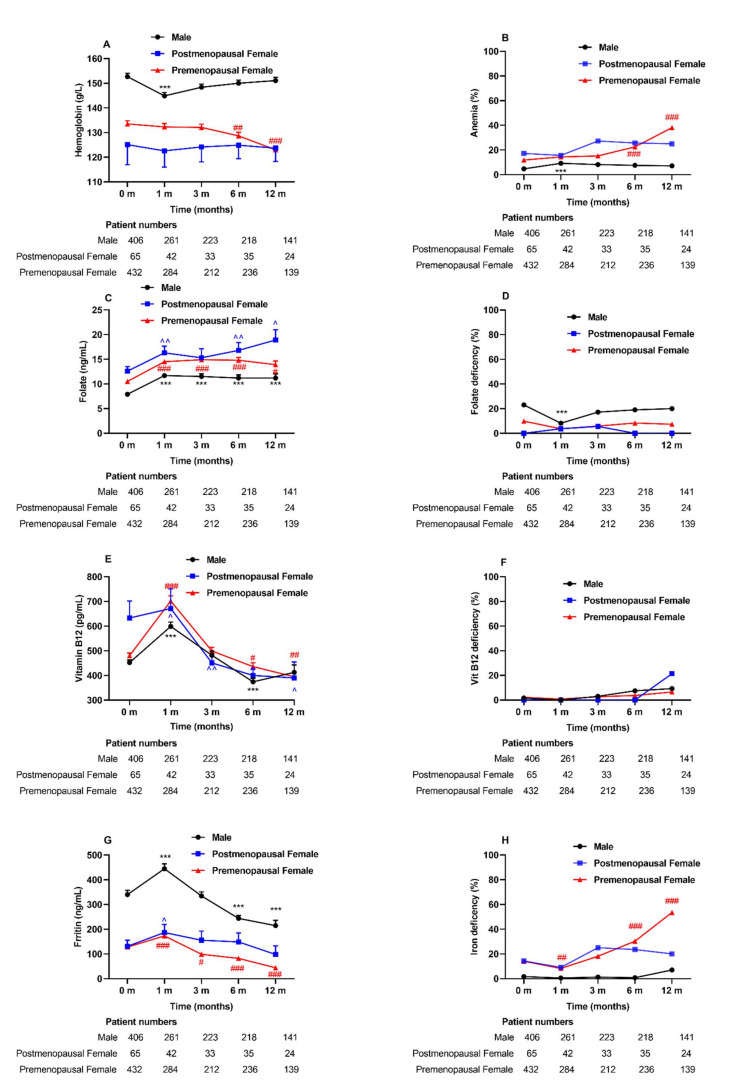
Anemia and related nutritional deficiencies during the 1-year follow-up after MS among three groups: male, postmenopausal, and premenopausal females. Abbreviations: MS, metabolic surgery. Data are presented as the mean ± SEM. *** *p* < 0.001 for each visit time compared with baseline in male subjects. ^ *p* < 0.05, ^^ *p* < 0.01 for each visit time compared with baseline in postmenopausal female subjects. # *p* < 0.05, ## *p* < 0.01, ### *p* < 0.001 for each visit time compared with baseline in premenopausal female subjects.

In the premenopausal female group, the Hb levels decreased at 6–12 months postsurgery compared with the baseline Hb levels (Figure 2A). Simultaneously, only in the premenopausal female group did anemia prevalence increase significantly at 12 months postoperation (11.9% vs. 38.1%, *p* = 0.000) (Figure 2B). For the male group, there was a temporary decrease in Hb levels and an increase in anemia prevalence at the 1-month visit after surgery. At the last visit, anemia was present in 69 patients (42, mild; 23, moderate; and 4, severe); 10 (14.5%) were male patients, and 59 (85.5%) were female patients. Moreover, multiple logistic regression indicated that preoperative anemia was associated with a higher risk of suffering anemia 1 year after MS. The odds ratio was 18.75 (95% CI, 2.1 to 167.6) after adjustment for sex, age, BMI, surgery type, and the deficiency status of folic acid, vitamin B12, and iron.

The folate levels were increased postoperatively (Figure 2C), although no significant difference in the folate deficiency rate was observed in all patients after 1-year visits. (Figure 2D). Interestingly, vitamin B12 levels increased at 1 month (*p* < 0.001) after surgery and then decreased at the following visits (Figure 2E). Nevertheless, the prevalence of vitamin B12 deficiency remained unchanged at 12 months after surgery (Figure 2F). Among all patients, the ferritin levels increased significantly at 1 month after surgery and then decreased at the following visits (Figure 2G). The iron deficiency (defined as ferritin levels <30 ng/mL) was increased in the premenopausal female groups across our study (14.1% vs. 53.5%, *p* = 0.000) (Figure 2H).

### 3.3. Bone Metabolism

**Figure 3:**
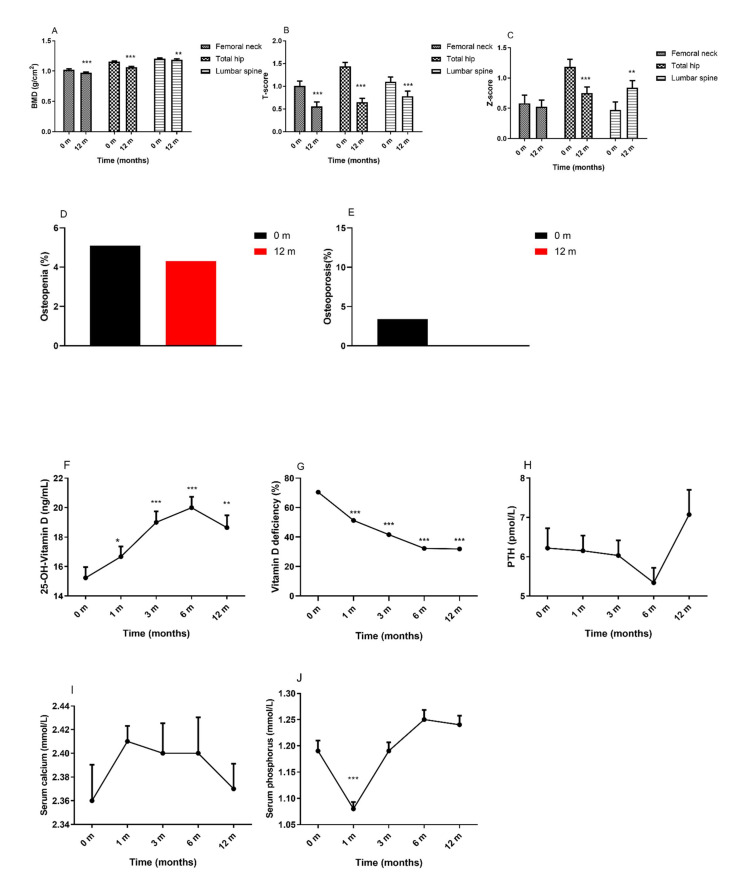
Changes in bone metabolism and BMD in patients following MS. (**A**): BMD; (**B**): T-score; (**C**): Z-score; (**D**): Osteopenia (%); (**E**): Osteoporosis (%); (**F**): 25-OH-Vitamin D; (**G**): Vitamin D deficiency (%); (**H**): PTH; (**I**): Serum calcium; (**J**): Serum phosphorus; Abbreviations: BMD, bone mineral density; MS, metabolic surgery; PTH, parathyroid hormone. Data are presented as the mean ± SEM. * *p* < 0.05, ** *p* < 0.01, *** *p* < 0.001 compared with baseline.

As shown in Figure 3A, overall, the BMD of the femoral neck, total hip, and lumbar spine significantly decreased at 12 months after surgery. The female subjects and the subjects who underwent RYGB showed decreases in the BMD of the femoral neck, total hip, and lumbar spine (Supplementary Tables S2 and S3). In Figure 3B, the T-scores of the femoral neck, total hip, and lumbar spine also decreased at 12 months after surgery, and we also found significant decreases in the Z-scores of the total hip (Figure 3C). In Figure 3D, 5.1% of patients were diagnosed with osteopenia at baseline, and 4.3% were diagnosed with osteopenia after surgery. In Figure 3E, 3.4% were diagnosed with osteoporosis preoperatively, while no patients were diagnosed with osteoporosis postoperatively. Regardless of sex and surgery type, subgroup analysis revealed no significant alterations in the prevalence of osteopenia and osteoporosis postoperatively (Supplementary Tables S2 and S3).

Vitamin D and parathyroid hormone (PTH) are the key regulators of calcium homeostasis in the body and play an important role in bone metabolism. The 25-OH-vitamin D levels increased at 12 months after surgery (Figure 3F), while approximately one-third of subjects showed 25-OH-vitamin D deficiency after surgery (Figure 3G). The levels of PTH (Figure 3H) and serum calcium (Figure 3I) were not significantly different from baseline to the 12-month visit. Serum phosphorus (Figure 3J) levels decreased at 1 month and then increased to baseline levels at the following visits. For patients in the female and RYGB groups, the levels of PTH and serum phosphorus increased significantly from baseline to the 12-month visit. Patients in the male and SG groups showed increased levels of 25-OH-vitamin D and serum calcium postoperatively (Supplementary Tables S2 and S3).

## 4. Discussion

During the 1-year follow-up, MS has resulted in a markable weight loss for patients with obesity, while there was no significant difference in weight loss between SG and RYGB. The Strasbourg RCT reported that weight-loss outcomes were similar in the two surgical groups at 1 year, while RYGB resulted in more stable weight loss than SG at 5 years [30]. Furthermore, a meta-analysis reported that RYGB and SG achieve comparable weight loss within the first postoperative year, but RYGB may provide greater weight loss at 2–5 years postoperatively than SG [31]. It seems that the difference in weight loss between the two surgeries increased with time. Compared with most US studies, the patients in our study were younger and weighed less, so the difference in the patient populations that were studied may have contributed to the difference in results [32,33]. The SM-BOSS RCT that was conducted in Switzerland also reported similar weight loss between the two procedures at 5 years [5]. Thus, different patient populations may contribute to this discrepancy. Our study found that patients in the RYGB group had an excessive decrease in LDL-C levels compared to the SG group at the 1-year follow-up, which is similar to the results of other studies [34,35]. In our study, patients who underwent SG were younger and heavier but had mild metabolic disorders compared with patients who underwent RYGB. Overall, SG is easier to perform and has a shorter operation time. Additionally, SG has fewer complications compared with RYGB, which is suitable for obese patients with mild or without metabolic disorders.

Although studies have confirmed the metabolic benefits of MS, investigators should pay more attention to the nutritional status of patients with obesity following surgery. For patients accepted MS, reduced mechanical digestion and acid secretion impairs the digestion and absorption of iron, vitamin B12, and other nutrients. Additionally, the diminished secretion of intrinsic factor results in further impairment of vitamin B12 absorption. Furthermore, decreased secretion of ghrelin contributes to appetite suppression and low nutrition intake [36]. Patients are prone to develop nutritional deficiencies due to caloric intake restriction and/or gastrointestinal tract alterations after MS. To our knowledge, this is the largest study assessing nutritional status following MS in Chinese patients and the only multicenter cohort study.

The incidence of anemia following MS has been reported to vary widely across studies because of the complexity of influencing factors, such as different populations, operation types, and supplement protocols. In the current study, we noted that Hb levels decreased at 12 months, especially in premenopausal female and male subjects (Figure 2A). While a significant increase was noted in the incidence of anemia in premenopausal females following surgery (Figure 2B), fortunately, among patients with anemia, approximately 94.2% were diagnosed with mild or moderate anemia, which is consistent with the results of a French survey [37], indicating that female sex is an independent factor in predicting anemia after RYGB. There was evidence demonstrating significantly higher rates of anemia at 24–48 months postoperation compared to preoperative rates (23% vs. 12%) in America after RYGB [14].

Iron deficiency is generally related to microcytosis, while vitamin B12 and folate deficiency are generally related to macrocytosis. In our study, the ferritin levels increased significantly at 1 month after surgery and then decreased at the following visits (Figure 2G). When interpreting the iron status, systemic inflammation needs to be considered, as it can significantly alter serum iron metabolism [38]. The inflammatory markers, such as IL-6 and C-reactive protein (CRP), decreased after MS [39], along with decreased serum transferrin receptor levels [40]. Furthermore, IL-6 upregulates hepcidin, a peptide that decreases iron absorption and releases iron from stores [41]. The significant decrease in the serum iron level at 1 month postoperative could be ascribed to the increased inflammation relatively soon after surgery. The improvement in serum iron found in our study might imply an improvement in iron bioavailability through decreased inflammation. The low levels of ferritin and iron indicate iron deficiency, which is an important cause of anemia. Iron deficiency after MS. Otherwise, iron deficiency in MS patients may arise from a reduced bioavailability of dietary iron because of the lack of hydrochloric acid production in the small gastric pouch. In addition to be a nutrition index, ferritin is also an acute-phase reactant which can be synthesized by multiple cytokines stimulation [27]. Recently, a low-grade, chronic inflammation status, which contributes to insulin resistance and T2D, has been proven to be associated with obesity [42]. MS alleviates the inflammatory status in subjects with obesity [39]. Therefore, the decline in serum ferritin after MS represents a reduction in body iron stores and an improvement in inflammation. A significant increase was noted in iron deficiency in the premenopausal female group across our study (Figure 2H), which suggested that iron deficiency is associated with anemia postoperation.

Vitamin B12 and folate are necessary to sustain normal hematopoiesis. Vitamin B12 and/or folate deficiencies will cause megaloblastic anemia. In our study, vitamin B12 levels increased at 1 month after surgery and then decreased at the following visits (Figure 2E). We also found no changes in vitamin B12 deficiency in all subjects at 12 months after MS. The reduction of intrinsic factors contributes to vitamin B12 deficiency after MS [43]. A significant decrease in vitamin B12 deficiency five years after LSG in Canada was demonstrated [44], in which the subjects were instructed to take 20–40 μg of vitamin B12 daily [44]. Nutrient supplements are considered sufficient and important postoperation. The primary reason for folate deficiency is decreased dietary intake, as folate absorption can occur along the entire part of the small intestine. Our study reported increased level of folate (Figure 2C), and others demonstrated an improvement in folate deficiencies after laparoscopic RYGB (LRYGB) in America and in Denmark [14,45].

Studies have reported the effect of MS on bone metabolism. The evidence showed that weight loss interventions led to increased bone loss over time, although there was discordance between the different kinds of methods for BMD analysis. The skeletal effects of MS are also partly dependent on the type of surgical procedure. RYGB was found to cause significantly greater bone loss than sleeve gastrectomy (SG) [46]. Our study showed that the total hip, spine and femoral neck BMDs measured by DEXA were decreased in patients after RYGB and SG. Being overweight and underweight are associated with certain types of fractures in postmenopausal women, which suggests that maintaining a normal BMI is important for the prevention of bone loss [47]. After MS, weight reduction decreases mechanical loading on bone, which induces mechanical strain on the skeleton that is associated with the BMD preservation [48]. Simultaneously, elevated serum concentrations of bone formation and resorption markers after MS were reported, suggesting an increase in bone remodeling in patients after MS [49]. The increase in bone resorption markers exceeds the increase in bone formation, which results in net bone loss [50].

At 12 months after RYGB or SG, our study showed that the mean serum calcium, phosphorus, and PTH levels were unchanged, while the 25-OH-vitamin D levels were increased in the patients. Within the three-year follow-up, patients showed normal calcium and phosphorus plasma concentrations and decreased 25-OH-vitamin D levels. Moreover, the BMDs at the femoral neck and lumbar spine were significantly decreased [51]. A negative correlation was described between 25-OH vitamin D and PTH levels in women after MS [52,53], which suggests that vitamin D supplementation partly improves secondary hyperparathyroidism. Even clinical studies have reported declines in BMD and increases in bone turnover markers without changes in circulating calcium, vitamin D or PTH levels [54]. Proper supplementation of vitamin D, calcium, and protein are still encouraged for patients after MS to prevent bone loss. After nutritional supplementation, patients showed less decline in the BMD of the lumbar spine, total hip and total body, as well as trabecular bone score (TBS) values after RYGB or SG. Moreover, the increases in bone turnover markers are less pronounced, which indicates that supplementations are beneficial to the bone metabolism after MS and should therefore be recommended for all patients accepting this surgery [55]. It is necessary to treat vitamin D deficiency in overweight and obese patients with alternative regimens. Compared with normal weight individuals, obese individuals have lower vitamin D levels, and even when supplemented with the same dose of vitamin D, nonobese children and adults, compared to obese children, have higher serum levels of vitamin D, possibly due to sequestration of vitamin D in body fat stores [56]. Despite the observed bone loss following MS, the data do not conclusively support an increased incidence of osteoporosis in patients after MS [57]. Overall, our study also found no patients with newly diagnosis of osteoporosis; hence, further research is needed to provide conclusive evidence with a long-term follow-up.

To sum up, our study evaluated nutritional status following MS in Chinese patients. During the 1 year follow-up, our study revealed that the prevalence of anemia and bone loss increased significantly in Chinese patients after MS.

## 5. Conclusions

In summary, our study demonstrate that RYGB exhibited a superior effect on lipid disorders compared to SG. Furthermore, based on the 12-month visits, we found that anemia prevalence increased significantly at 12 months postoperation in premenopausal females, which is probably associated with iron deficiency. Moreover, patients with preoperative anemia have an increased risk of postoperative anemia. Although the BMD of the femoral neck, total hip, and lumbar spine significantly decreased postoperatively, no new patients developed osteoporosis. Female sex and RYGB are related to anemia and bone loss. Recognition of these postoperative deficiencies will remain an ongoing educational process. Further studies are needed to examine the standardized pre- and postoperative measurements and to determine the appropriate supplemental prescriptions following MS.

## Figures and Tables

**Table 1:** Anthropometric and clinical characteristics of the study subjects at baseline.

	All	RYGB	SG	*p*-Value (RYGB vs. SG)
N	903	263	640	/
Age (years)	31 (26–40)	37 (29–47)	30 (24–36)	<0.001
Sex (female/male)	497/406	136/127	361/278	0.189
BMI (Kg/m^2^)	37.7 (33.7–42.6)	37.2 (31.2–42.6)	38.0 (34.3–42.6)	0.004
Waist circumference (cm)	117 (107–129)	115 (103–129)	118 (108–130)	0.006
Hip circumference (cm)	119 (110–129)	116 (105–128)	120 (112–130)	<0.001
SBP (mmHg)	134 (124–148)	136 (123–149)	133 (124–147)	0.200
DBP (mmHg)	84 (77–92)	85 (77–93)	84 (77–92)	0.286
FPG (mmol/L)	5.6 (4.9–7.2)	6.4 (5.4–9.2)	5.3 (4.8–6.5)	<0.001
HbA1c (%)	6.0 (5.5–7.3)	7.1 (5.9–8.5)	5.8 (5.5–6.6)	<0.001
TG (mmol/L)	1.8 (1.3–2.5)	2.0 (1.4–2.8)	1.7 (1.2–2.4)	<0.001
TC (mmol/L)	4.6 (3.9–5.2)	4.7 (4.1–5.4)	4.5 (3.9–5.2)	0.036
HDL-C (mmol/L)	1.0 (0.8–1.1)	1.0 (0.8–1.1)	1.0 (0.8–1.1)	0.094
LDL-C (mmol/L)	2.8 (2.3–3.4)	2.8 (2.3–3.3)	2.8 (2.3–3.4)	0.351

Abbreviations: RYGB, Roux-en-Y gastric bypass; SG, sleeve gastrectomy; BMI, body mass index; SBP, systolic blood pressure; DBP, diastolic blood pressure; HbA1c, hemoglobin A1c; FPG, fasting plasma glucose; TG, triglyceride; TC, total cholesterol; HDL-C, high-density lipoprotein cholesterol; LDL-C, low-density lipoprotein cholesterol. the mean ± SD or the median (25–75th percentile).

**Table 2:** Case-matched study of weight loss and metabolic control between RYGB and SG.

	RYGB (*n* = 98)	SG (*n* = 98)	*p*-Value (RYGB vs. SG)
Gender (female/male)	52/46	60/38	0.312
Age (years)	33 (28–42)	33 (28–41)	0.800
Preop BMI (kg/m^2^)	38.6 ± 5.7	38.2 ± 5.8	0.929
1-year BMI (kg/m^2^)	27.6 ± 3.6 ***	26.7 ± 4.7 ***	0.384
1-year EBMIL (%)	81.2 (67.5–102.5)	91.1 (72.0–128.4)	0.127
Preop FPG (mmol/L)	5.7 (5.0–6.6)	5.5 (4.9–6.6)	0.872
1-year FPG (mmol/L)	4.9 (4.5–5.4) ***	4.5 (4.2–5.0) ***	0.684
Preop PPG (mmol/L)	8.8 (7.0–12.2)	8.8 (6.4–13.1)	0.493
1-year PPG (mmol/L)	4.7 (4.3–5.1) ***	5.0 (4.1–5.6) ***	0.303
Preop HbA1c (%)	6.0 (5.6–6.7)	5.9 (5.6–6.5)	0.592
1-year HbA1c (%)	5.3 (5.0–5.6) ***	5.4 (5.1–5.6) ***	0.620
Preop TG (mmol/L)	1.9 (1.4–2.6)	1.5 (1.2–2.2)	0.088
1-year TG (mmol/L)	0.8 (0.7–1.2) **	0.9 (0.7–1.2) ***	0.638
Preop TC (mmol/L)	4.7 ± 0.9	4.7 ± 0.9	0.918
1-year TC (mmol/L)	4.1 ± 0.8 ***	4.5 ± 1.1	0.063
Preop HDL-C (mmol/L)	0.9 (0.8–1.1)	1.0 (0.9–1.1)	0.805
1-year HDL-C (mmol/L)	1.4 (1.2–1.6) ***	1.4 (1.1–1.6) ***	0.876
Preop LDL-C (mmol/L)	2.8 ± 0.7	3.0 ± 0.8	0.300
1-year LDL-C (mmol/L)	2.2 ± 0.5 ***	2.7 ± 1.0	0.008
Preop SBP (mmHg)	136 (124–148)	132 (122–145)	0.157
1-year SBP (mmHg)	117 (110–128) ***	124 (110–127)	0.782
Preop DBP (mmHg)	86 (78–94)	82 (75–90)	0.156
1-year DBP (mmHg)	75 (67–86) ***	74 (68–80) *	0.329

Abbreviations: RYGB, Roux-en-Y gastric bypass; SG, sleeve gastrectomy; BMI, body mass index; EBMIL, excess body mass index loss; FPG, fasting plasma glucose; PPG, postprandial plasma glucose; HbA1c, hemoglobin A1c; TG, triglyceride; TC, total cholesterol; HDL-C, high-density lipoprotein cholesterol; LDL-C, low-density lipoprotein cholesterol. SBP, systolic blood pressure; DBP, diastolic blood pressure. Data are presented as the mean ± SD or the median (25–75th percentile). * *p* < 0.05, ** *p* < 0.01, *** *p* < 0.001 compared with baseline.

## Data Availability

Data is contained within the article or supplementary material.

## Supplementary Materials

The following supporting information can be downloaded at: https://www.mdpi.com/article/10.3390/nu14091932/s1, Table S1: Changes in clinical parameters during the 1-year follow-up; Table S2: Variations in clinical data and bone mineral density of the female and male subjects following MS; Table S3: Variations in clinical data and bone mineral density of subjects following RYGB or SG
