# Supplementary material for: Nutritional Status in Chinese Patients with Obesity Following Sleeve Gastrectomy/Roux-en-Y Gastric Bypass: A Retrospective Multicenter Cohort Study
Source: Nutrients. 2022 May 5;14(9):1932. doi: 10.3390/nu14091932 (PMC9101375; doi:10.3390/nu14091932)
Supplement: Supplementary file 1 [file nutrients-14-01932-s001.zip › nutrients-1670076-SI.pdf]

Supplementary table S1: Changes in clinical parameters during the 1-year follow-up.

	Baseline	12m
BMI(Kg/m^2)	37.9 (33.4-42.9)	26.9 (23.9-30.3) ***
EBMIL (%)	/	84 (67-107)
Waist Circumference (cm)	115 (105-126)	92 (84-98) ***
Hip Circumference (cm)	117 (107-129)	100 (94-106) ***
SBP (mmHg)	132 (123-146)	118 (109-129) ***
DBP (mmHg)	84 (76-91)	74 (67-83) ***
FPG (mmol/L)	6.0 (5.2-8.1)	4.6 (4.3-5.1) ***
HbA1c (%)	6.3 (5.5-7.8)	5.3 (5.1-5.6) ***
TG (mmol/L)	1.8 (1.2-2.6)	0.9 (0.7-1.2) ***
TC (mmol/L)	4.7±1.0	4.3±0.9 ***
HDL-C (mmol/L)	1.0 (0.8-1.1)	1.3 (1.1-1.5) ***
LDL-C (mmol/L)	2.8±0.8	2.5±0.8 ***
DM/ Pre-DM (%)	74.8	15.8 ***
High blood pressure (%)	58.5	21.6 ***
Dyslipidemia (%)	73	21.3 ***

Abbreviations: RYGB, Roux-en-Y gastric bypass; SG, sleeve gastrectomy; BMI, body mass index; EBMIL, excess body mass index loss; FPG, fasting plasma glucose; PPG, postprandial plasma glucose; HbA1c, hemoglobin A1c; TG, triglyceride; TC, total cholesterol; HDL-C, high-density lipoprotein cholesterol; LDL-C, low-density lipoprotein cholesterol. SBP, systolic blood pressure; DBP, diastolic blood pressure.

Data are presented as the mean ± SD or the median (25th–75th percentile). ***P<0.001 compared with baseline.

Supplementary Table S2: Variations in clinical data and bone mineral density of the female and male subjects following MS.

	Baseline		12 Months	
	Female	Male	Female	Male
Age (years)	34 (29-43)	32 (27-45)	\	\
BMI (kg/m^2)	37.0 (33.6-41.9)	39.0 (33.0-44.0) ***	27.5 (23.3-29.9) $^{###}$	26.3 (24.7-30.4) $^{+++}$
EBMIL (%)	\	\	79.9 (64.7-101.4)	89.0 (68.1-118.1) *
BMD in Femoral neck (g/cm^2)	1.00±0.13	1.04±0.17***	0.95±0.13$^{###}$	0.99±0.16^{+++}
BMD in Total hip (g/cm^2)	1.13±0.12	1.17±0.14**	1.05±0.12$^{###}$	1.07±0.14^{+++}
BMD in Lumbar spine (g/cm^2)	1.21±0.15	1.15±0.18*	1.19±0.16$^{#}$	1.14±0.16
Osteopenia (%)	5.8	2.2	2.9	4.3
Osteoporosis (%)	4.3	2.2	0	0
25-OH-Vitamin D (ng/mL)	16.1 (11.5-26.2)	21.1 (12.7-34.8) **	20.8 (12.7-28.0)	23.9 (16.6-37.5) $^{++}$
Vitamin D deficiency (%)	61.5	45.9*	46.2$^{#}$	37.7
PTH (pmol/L)	5.0 (3.3-8.0)	5.9 (3.8-7.9)	6.2 (3.9-9.3) $^{#}$	6.4 (4.1-9.7)
Serum calcium (mmol/L)	2.3 (2.2-2.4)	2.3 (2.2-2.4)	2.3 (2.2-2.4)	2.4 (2.3-2.5) $^{+++}$
Serum phosphorus (mmol/L)	1.2 (1.1-1.3)	1.3 (1.1-1.3)	1.3 (1.2-1.4) $^{##}$	1.2 (1.1-1.4)

Abbreviations: MS, metabolic surgery; BMI, body mass index; EBMIL, excess body mass index loss; BMD, bone mineral density; PTH, parathyroid hormone.

Data are presented as the mean ± SD and the median (25th–75th percentile). * $P < 0.05$, ** $P < 0.01$, *** $P < 0.001$ in the female group compared with the male group at baseline. $^{\#}$ $P < 0.05$, $^{\#\#}$ $P < 0.01$, $^{\#\#\#}$ $P < 0.001$ compared with baseline in the female group. $^{++}$ $P < 0.01$, $^{+++}$ $P < 0.001$ compared with baseline in the male group.

Supplementary Table S3: Variations in clinical data and bone mineral density of subjects following RYGB or SG.

	Baseline		12 Months	
	RYGB	SG	RYGB	SG
Age (years)	34 (28-44)	30 (24-36) ***	\	\
BMI (kg/m^2)	36.2 (30.7-41.2)	38.7 (34.7-43.2) **	26.8 (23.9-30.1) ###	26.8 (23.8-30.6) +++
EBMIL (%)	\	\	80.8 (63.9-107.1)	87.1 (67.6-107.6)
BMD in Femoral neck (g/cm^2)	1.03±0.15	1.02±0.15*	0.96±0.14###	0.98±0.15+++
BMD in Total hip (g/cm^2)	1.17±0.13	1.15±0.13*	1.07±0.13###	1.07±0.13+++
BMD in Lumbar spine (g/cm^2)	1.28±0.17	1.16±0.16***	1.24±0.18###	1.16±0.16
Osteopenia (%)	10.0	1.3	7.5	1.3
Osteoporosis (%)	7.5	1.3	0	0
25-OH-Vitamin D (ng/mL)	13.1 (9.7-19.2)	25.9 (15.3-37.3) ***	16.6 (10.7-21.7)	30 (21.3-42.5) +
Vitamin D deficiency (%)	78.9	33.3	66.7#	21.7
PTH (pmol/L)	6.0 (3.1-8.0)	4.9 (4.3-8.0)	6.8 (4.0-9.8) #	5.3 (3.9-8.1)
Serum calcium (mmol/L)	2.4 (2.2-2.5)	2.3 (2.2-2.4)	2.4 (2.3-2.5)	2.3 (2.3-2.4) ++
Serum phosphorus (mmol/L)	1.2 (1.1-1.3)	1.2 (1.1-1.3)	1.3 (1.1-1.4) #	1.2 (1.2-1.3)

Abbreviations: RYGB, Roux-en-Y gastric bypass; SG, sleeve gastrectomy; BMI, body mass index; EBMIL, excess body mass index loss; BMD, bone mineral density; PTH, parathyroid hormone.

Data are presented as the mean ± SD and the median (25th–75th percentile). * $P < 0.05$, ** $P < 0.01$, *** $P < 0.001$ in the RYGB group compared with the SG group at baseline. $^{\#}$ $P < 0.05$, $^{\#\#\#}$ $P < 0.001$ compared with baseline in the RYGB group. $^{+}$ $P < 0.05$, $^{++}$ $P < 0.01$, $^{+++}$ $P < 0.001$ compared with baseline in the SG group.
